# Hydrostatic Pressures in Lymphatic Networks of the Mouse

**DOI:** 10.1111/micc.70046

**Published:** 2025-12-18

**Authors:** Michael J. Davis

**Affiliations:** ^1^ Department of Medical Pharmacology & Physiology University of Missouri Columbia Missouri USA

**Keywords:** mesentery, popliteal, servo‐null micropressure, superficial cervical

## Abstract

**Objective:**

Current research on the lymphatic system makes nearly exclusive use of mouse models because that species is highly amenable to genetic manipulation. However, the lack of information about intraluminal hydrostatic pressures in mouse lymphatic networks limits the ability of investigators, for both in vivo and ex vivo studies, to interpret key physiological data. The goal of the present study was to provide some of that information for several commonly studied lymphatic vessel networks of young, healthy mice: the superficial cervical lymphatic network, the popliteal afferent network, and the mesentery network.

**Methods:**

The servo‐null micropressure method was used to measure intraluminal hydrostatic pressures in lymphatic vessels, taking advantage of *Prox1‐GFP* and *Prox1‐tom* mice, expressing eGFP and td‐tomato reporters, respectively, in lymphatic endothelium, to visualize entire lymphatic networks and facilitate micropuncture of individual vessels.

**Results:**

New methods were devised for making servo‐null pressure measurements under fluorescence illumination. Intraluminal pressures oscillated with the lymphatic contraction cycle, and systolic pressure peaks coincided with lymphatic systole. The largest peaks occurred when contractions of consecutive lymphangions were entrained. In networks with active contractions, the pressure profile was “uphill”; in networks without contractions, the pressure profile was “downhill.”

**Conclusions:**

Our results provide essential information about the “normal” values for intraluminal hydrostatic pressures in several of the most commonly studied lymphatic networks of the mouse.

## 1 Introduction

The lymphatic system is a one‐way vascular network responsible for reabsorbing excess interstitial fluid and protein and returning it to the central veins. The daily transport of lymph may exceed 8 L of fluid and 120 g of protein per day in humans [1]. Although lymphatic networks are almost universally considered to be low pressure systems, limited information has been published on the values of hydrostatic pressures in lymphatic vessels in vivo and even less on the pressure distributions across lymphatic vascular branching orders—capillaries, precollectors, and collectors—within a given network. For example, Hargens and Zweifach [2, 3] used the servo‐null micropressure technique to measure hydrostatic pressures throughout mesenteric lymphatic networks of several species, and the depiction of representative pressure recordings in sequential branching orders of lymphatic vessels in a review article by Zweifach and Lipowsky [4] led to the now‐established concept that luminal pressures are stable at or near atmospheric levels (i.e., zero gauge pressure) in lymphatic capillaries [5] but gradually rise and become pulsatile in actively pumping collectors, reaching pressure levels of ~10–15 cmH_2_O near the mesenteric lymph node.

Multiple investigators have measured hydrostatic pressures in individual lymphatic vessels in vivo. For example, Benoit et al. [6] reported end‐diastolic pressures of 1.7 cmH_2_O in mesenteric collectors of the rat that increased to 3.8 cmH_2_O after systemic volume expansion. In villus capillaries of the rat small intestine, Lee [7] recorded pressures of 1.4 cmH_2_O in isotonic Na^+^ solution that increased to 5.2 cmH_2_O in hypotonic Na^+^ solution (simulating a state of post‐prandial reabsorption). In a notable study, Unthank and Hogan simultaneously measured pressures inside and outside the lymphatic capillary lumen in bat wing—a species in which initial lymphatics are inherently contractile. Their results were critical for establishing the concept that phasic contractions generate cyclic fluctuations in luminal pressure to create a favorable hydrostatic pressure gradient for reabsorption of lymph during the diastolic phase of the contraction cycle [8, 9]. The principle of a “suction effect” that results from the expansion of the vessel wall during diastole and potentially facilitates reabsorption was subsequently confirmed in collecting lymphatic vessels [10]. Fluctuating pressure gradients also determine reabsorption in lymphatic networks of the diaphragm during the respiratory cycle [11, 12, 13].

In larger animals, lymphatic pressures have typically been determined by direct cannulation. Measurements in lymphatic collectors of dogs and sheep [14, 15] led to the concept that post‐nodal pressures are lower than prenodal pressures but gradually increase prior to the next downstream node due to active contractions of the collecting vessels [16]. Olszewski directly cannulated human lymphatics in patients that were either healthy or in various stages of lymphedema [17, 18, 19] and found (1) that collecting vessels in the lower leg are capable of generating peak systolic pressures > 60 mmHg (82 cmH_2_O) to overcome adverse gravitational pressure gradients (an exception to the concept of universally low pressure lymphatic networks); and (2) that diastolic lymphatic pressures become elevated in the legs of upright lymphedema patients if the valves are incompetent.

In recent years, lymphatic research has turned increasingly to the use of mouse models, due to the ease with which that species can be genetically manipulated and with the widespread availability of transgenic mice. However, direct measurements of lymphatic pressures in the mouse are lacking, except for an older report in mouse ear and a recent report of pressures in mesenteric collecting vessels of the terminal ileum. In initial lymphatics of the mouse the ear, pressures were 1–2 cmH_2_O [20] and in mesenteric collectors, pressures averaged ~4 cmH_2_O in control mice but ~14 cmH_2_O upstream from tertiary lymphoid organs that develop in/around the collectors, impeding lymphatic outflow in animals that overexpress TNFα [21]. The lack of direct pressure measurements in other lymphatic networks of the mouse has limited the ability of investigators using pressure myography to determine the appropriate pressure levels for ex vivo studies of lymphatic vessels [22]. Likewise, in vivo studies making comparisons between control and genetically modified mice or between mice with/without experimental lymphedema must assume that lymphatic pressure changes do not contribute to any observed differences in transport or contractile function [23, 24, 25, 26, 27, 28, 29]; yet those assumptions have not been verified experimentally.

A need therefore exists for fundamental information on vascular hydrostatic pressures in mouse lymphatic networks. The goal of the present study was to provide such information for three of the most studied lymphatic vessel networks of young, healthy mice: the superficial cervical lymphatic network, the popliteal afferent network, and the mesentery network. Due to the small sizes of peripheral lymphatic vessels in mice, we used the “gold‐standard” for measurement of microvascular hydrostatic pressure: the servo‐null micropressure method [30, 31]. We took advantage of *Prox1‐GFP* and *Prox1‐tom* mice, expressing fluorescence reporters selectively in lymphatic endothelium, to visualize entire lymphatic networks and facilitate the micropuncture of individual vessels—a task that otherwise would have been difficult in some body regions. It was first necessary to develop new methods for making servo‐null measurements under fluorescence illumination. Our results allow us to establish the “normal” values of hydrostatic pressure in three commonly studied lymphatic networks of the mouse as well as to compare the pressure distributions in passive and actively contracting lymphatic vessel networks.

## 2 Methods

### 2.1 Mice

All animal procedures were approved by the animal care committee at the University of Missouri (protocol #9797) and complied with the standards stated in the “Guide for the Care and Use of Laboratory Animals” (National Institutes of Health, revised 2011). *Prox1‐GFP* mice were a gift from Young‐Kwon Hong, University of Southern California. C57Bl/6j (WT; #000664) mice and *Prox1‐tom* (#018128) mice were obtained from JAX (Bar Harbor, ME). Mice were anesthetized with an intraperitoneal injection of ketamine/xylazine (50/5 mg/kg). Supplemental anesthesia (at ½ that dose) was given subcutaneously as needed. Mice of either sex were studied at ages 3–8 months.

### 2.2 Solutions and Chemicals

Krebs solution contained: 146.9 mM NaCl, 4.7 mM KCl, 2 mM CaCl_2_·2H_2_O, 1.2 mM MgSO_4_, 1.2 mM NaH_2_PO_4_·H_2_O, 3 mM NaHCO_3_, 1.5 mM Na‐HEPES, and 5 mM D‐glucose (pH = 7.4). MgSO_4_ and Na‐HEPES were purchased from ThermoFisher Scientific (Pittsburgh, PA), and all other chemicals were obtained from Sigma‐Aldrich (St. Louis, MO) unless otherwise noted.

### 2.3 Superficial Cervical Lymphatics

With the mouse under anesthesia, superficial cervical lymphatic vessels were exposed through an incision on either side of the neck. The skin was retracted using small clamps (Roboz, #RS‐5470, #RS‐7500), and the exposed surface vessels were kept moist with Krebs solution. Loose connective tissue surrounding the vessel was removed using fine forceps and scissors until segments of the 3 main afferent lymphatic collectors feeding the submandibular node were exposed. Pressures in the SCLV1 and SCLV2 vessels [32] were measured either near the last valve before the vessel entered the lymph node (distal site), at a site 5–6 valves upstream from that site, where the vessels passed along the mandible (proximal site), or at an intermediate site. The preparation was superfused with Krebs solution fed by gravity from a reservoir and heated to 36°C–37°C through an in‐line solution heater (Harvard Apparatus #64‐0102) at a flow rate = 1–2 mL/min. The animal was heated from below by circulating heated water through a Lucite block with enclosed channels.

### 2.4 Popliteal Afferent Lymphatics

In an anesthetized mouse, the popliteal afferent lymphatic vessels were exposed through a superficial incision at one of three locations in the leg. The skin was retracted and held in place by small clamps anchored by dental wax, with the edges of the skin lifted slightly to permit the formation of a well of Krebs solution. The leg was extended with a slight bend and the tips of the toes were taped to hold it in a relatively natural position that permitted viewing and micropuncture of the afferent vessels. The two afferent popliteal collecting vessels on either side of the saphenous vein were identified and loose, overlying connective tissue was removed with fine scissors as needed to facilitate micropuncture. Three sites were used for micropuncture (but only one in each animal): (1) the mid‐calf region (a proximal site typically used for NIRF studies and for dissection of lymphatic collectors for ex vivo studies); (2) an intermediate site at the level of the ankle; and (3) the top of the foot where the collecting vessel network formed from capillaries and precollectors (distal site). To access the latter site, the mouse was placed on its back with the toes of one foot taped to the heated Lucite block. A 0.5–0.8 cm incision was made in the skin of the dorsal surface of the ipsilateral foot and the edges were held apart using clamps or sutures secured by tape to the block. The preparation was superfused with heated Krebs solution.

### 2.5 Mesenteric Lymphatic Network

For viewing the intestinal mesentery, a silicone platform (SYLGARD 184; Dow Corning, Midland, MI) was created to enable stabilization of the terminal ileum with fine pins (Fine Science Tools, #26002‐20). With the mouse under anesthesia, an incision was made along the abdominal midline to expose the cecum and terminal ileum, along with the attached mesentery. The cecum and a 4‐cm section of the terminal ileum were reflected out of the abdominal cavity and pinned to the platform, with care taken not to partially occlude the lymphatic outflow tract. The mesentery and gut wall were kept moist at 36°C–37°C with continuous perfusion of heated Krebs buffer. Both the pedestal and animal were heated from below. A dam of vacuum grease was placed around the edge of the skin flap to create a 1–3 mm deep layer of Krebs solution over the preparation, which drained through a Kimwipe wick and vacuum line. For some preparations, Krebs solution was supplemented with 1 μM isoproterenol (Tocris, #1747) to suppress movement of the gut wall [33].

### 2.6 Microscope System

All preparations were viewed under trans‐ and/or epi‐illumination using a Zeiss AxioZoom V12 macroscope fitted with an x‐y stage to which was clamped a micromanipulator assembly. A hybrid manual/hydraulic micromanipulator (Narishige, #MN‐2 and #MO‐202, respectively) controlled the position of the micropipette. During micropuncture, the light source was switched between brightfield and fluorescence as needed. To image the eGFP signal, Zeiss 488 nm excitation and 510–540 emission (FITC) filters were used. To image the td‐tomato signal, the Zeiss ds‐Red filter set was used. ZEN software (Zeiss) controlled the illumination and acquired images at 5 Hz from a Hamamatsu Flash 4 camera for off‐line analysis of vessel diameter.

### 2.7 Servo‐Null Measurements

Beveled, glass micropipettes (3–5 μm tips) containing 2 M NaCl (resistances = 1–3 MΩ) were used to micropuncture individual lymphatic vessels. Except as noted below, the pipettes were fashioned from borosilicate glass (1.0/0.5 mm, ID/OD; #30‐30‐1, Frederick Haer, Bowdoin, ME). Sharpening was performed either by beveling the tips on a rotating stone (Narishige, #EG‐44) or by controlled breakage of the tips to ~45° angle against a metal surface. Each pipette was connected to a servo‐null micropressure system (IPM model 5, La Jolla, CA), with a low‐pressure transducer (CyQ model 104, Nicholasville KY) used to measure the counterpressure generated by the hydraulic pump [34]. The ground connection was made using an Ag/AgCl wire placed under the open skin in the corner of the exposed preparation. The transducer was pre‐calibrated from 0 to 20 cmH_2_O using a moveable reservoir, with pressure referenced to atmospheric (zero gauge). The analog output of the system (servo‐null pressure, P_sn_) was connected to an A‐D interface (NI SCB‐68, National Instruments, Austin TX) and recorded at 30 Hz using a custom LabVIEW program (National Instruments).

**FIGURE 1:**
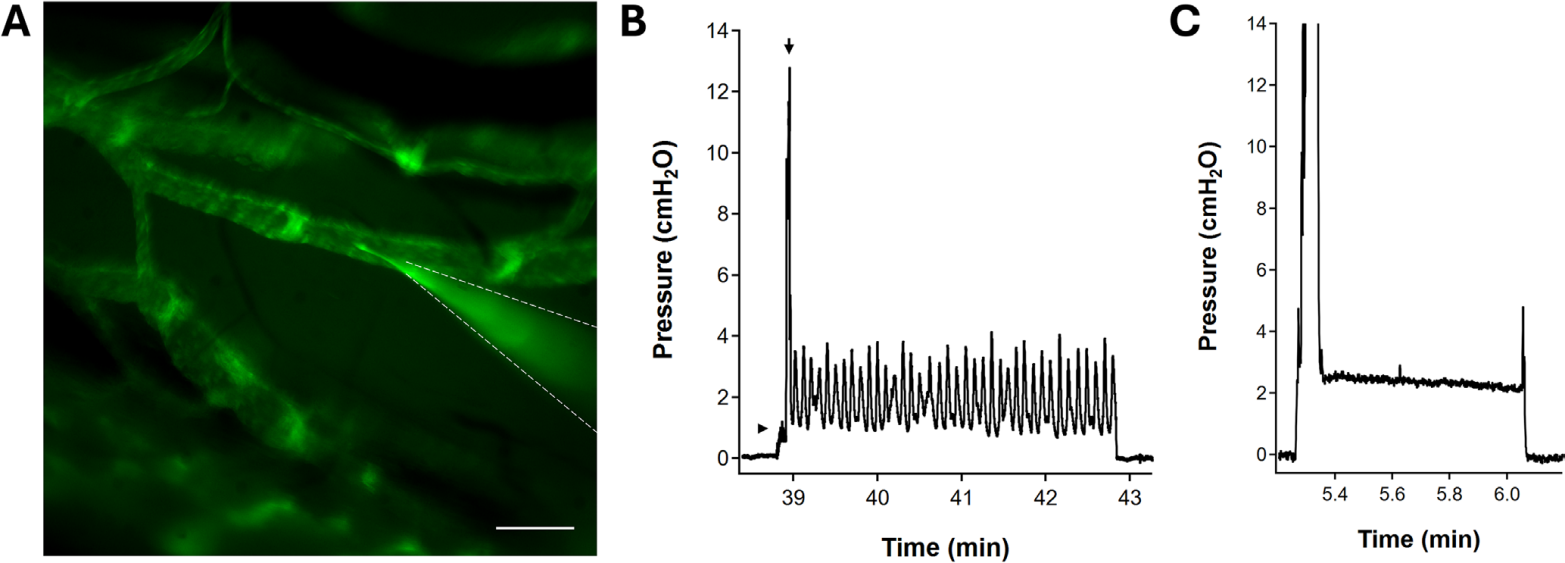
(A) Fluorescence image of a network of collectors and precollectors under the skin in the top of the foot of a *Prox1‐GFP* mouse, showing the tip of a servo‐null micropressure pipette in the lumen of a collecting lymphatic vessel. The tip of the pipette was painted with fluorescent dye to better visualize its position. The pipette shank is outlined with a dotted white line. The bright areas in the vessels are valves, including at least one venous valve. Scale bar = 200 μm. (B) Demonstration of changes in servo‐null micropressure signal before, during and after micropuncture in a superficial cervical collecting vessel. Pressure outside the vessel was 0 cmH_2_O and increased to ~1 cmH_2_O as the pipette was lowered gently onto the vessel surface (arrowhead). When the pipette was advanced through the vessel wall, pressure rose transiently to ~13 cmH_2_O (arrow) but then declined rapidly to a diastolic pressure of ~2 cmH_2_O as the tip was withdrawn slightly into an unobstructed position in the lumen. The pulsatile pressures reflect spontaneous contractions and associated luminal pressure (P_sn_) fluctuations. This pattern was stable over the subsequent 4 min period and slight side‐to‐side or up/down movements of the pipette (via the hydraulic manipulator) did not affect the P_sn_ recording, confirming that the tip was not touching the vessel wall. When the pipette was withdrawn, the P_sn_ signal returned to 0 cmH_2_O. (C) A similar recording in a mesenteric collector that did not exhibit spontaneous contractions.

The calibration of the transducer and servo‐null system, with a micropipette connected, was verified initially using an external calibration chamber as previously described [33]. Prior to each measurement, the zero level was set with the pipette tip positioned just outside the lymphatic vessel and checked again at the end of the recording after complete withdrawal of the pipette from the vessel lumen. In many experiments, visualization of the pipette tip under fluorescence illumination (using the FITC filter set) was aided by painting the tip with a fluorescent yellow dye (Sharpie) prior to sharpening/breaking (see Figure 1A). The pressure recording during wall penetration typically produced a pressure trace that rapidly rose to a relatively high value (> 20 cmH_2_O) as the tip advanced through the upper surface of the vessel wall (often touching the opposite wall) but then fell and stabilized as the pipette was partially withdrawn to leave the tip free in the vessel lumen (Figure 1B). Although pressure pulsations in synchrony with the heart rate are used in studies of arterioles and blood capillaries to confirm the position of the servo‐null pipette tip in the vessel lumen [33, 35], their absence in lymphatic vessels forced the development of other criteria for valid pressure measurements. In preparations exhibiting spontaneous contractions of collecting vessels, lymphatic pressures typically oscillated with diameter (as in Figure 1B), but this was not a definitive criterion (see exception below). An additional challenge in quiescent vessels (Figure 1C) was confirming that the tip was unobstructed in the lumen. In several initial experiments, the servo‐null micropipette was fashioned from two‐channel theta pipette tubing (Sutter Instruments, #BT150‐10) and a custom holder [36] allowing the connection of one pipette channel to the servo‐null system and the other to a glass syringe for injecting small volumes of Alexa543‐tagged albumin dissolved in Krebs solution. After micropipette tip placement in the vessel lumen, transient pressurization of the syringe produced puffs of dye that could be visualized in the ds‐Red fluorescence channel, confirming that the tip was unobstructed. When dye freely exited the tip, changes in the servo‐null feedback gain or small movements of the tip (using the hydraulic manipulator) did not alter the recorded P_sn_ value. In contrast, if the tip touched the vessel wall, the P_sn_ signal was often “noisy” and was altered by further movement or changes in the servo‐null gain. Likewise, if the tip touched the wall in a contracting vessel (or was only resting on the surface of the vessel), contraction‐induced P_sn_ spikes became exaggerated (up to 20 cmH_2_O in amplitude) upon slight lateral or vertical tip movement. However, theta tubing pipettes were used only in initial studies because the incidence of pipette plugging increased when the tagged albumin mixed with 2 M NaCl used for the servo‐null channel. Nevertheless, these preliminary tests enabled us to establish the following criteria for valid intraluminal pressure measurements: (1) accurate (±0.1 cmH_2_O) pre‐ and post‐measurement zero levels, (2) insensitivity of the servo‐null recording (±0.3 cmH_2_O) to changes in gain and to small imposed movements, (3) “clean” recordings free of mechanical noise (> 0.3 cmH_2_O) typically occurring when the pipette tip touched a vessel wall, and (4) pressure fluctuations in synchrony with spontaneous contractions (when present). When possible, two successive micropuncture recordings were attempted in each vessel.

### 2.8 Data Analysis

Pressure measurements in popliteal and superficial cervical vessels were usually obtained along with simultaneous diameter measurements from the same lymphangion (the latter made off‐line) for a sustained time period (1–10 min). Diameter tracking during playback of recorded fluorescence video (in AVI format) was performed with a modified version of a previously published program [37]. In reporter mice, the edge detection algorithms tracked the outside edges of fluorescent cells, that is, the outer edge of the endothelial cell layer, corresponding to the internal diameter of the vessel. Pressure and diameter signals (in LabVIEW and ZEN, respectively) were synchronized by periodically blanking the light source for the video while simultaneously marking a separate A‐D channel along with the digitized pressure recording and then aligning those signals during off‐line analysis. For popliteal and superficial cervical networks, the data were grouped according to location (proximal, distal, and intermediate, as appropriate). For the mesenteric network, measurements were recorded in as many different vessels as feasible for 1–2 min at each site (usually limited by the quality of the micropipettes and by intestinal wall movement). The data were then grouped according to vessel branching order: first, second, third order collectors, precollectors, or lymphatic capillaries, for statistical analysis.

### 2.9 Statistical Procedures

Microsoft Excel was used to compile the initial data, Prism (Graphpad v10; San Diego, CA) for summary plots/statistics, and Igor (Wavemetrics, Oswego, OR) to construct graphs of representative recordings and to align the pressure/diameter traces. One‐way ANOVAs with Tukey's post hoc tests were used to compare pressures between different branching orders of lymphatic vessels within a given region. Summary data are plotted as mean ± SEM. * indicates *p* < 0.05. Vessel numbers (*n*) and animal numbers (*N*) are shown or stated in the text and/or figure legends.

## 3 Results

### 3.1 Superficial Cervical Lymphatic Pressures

**FIGURE 2:**
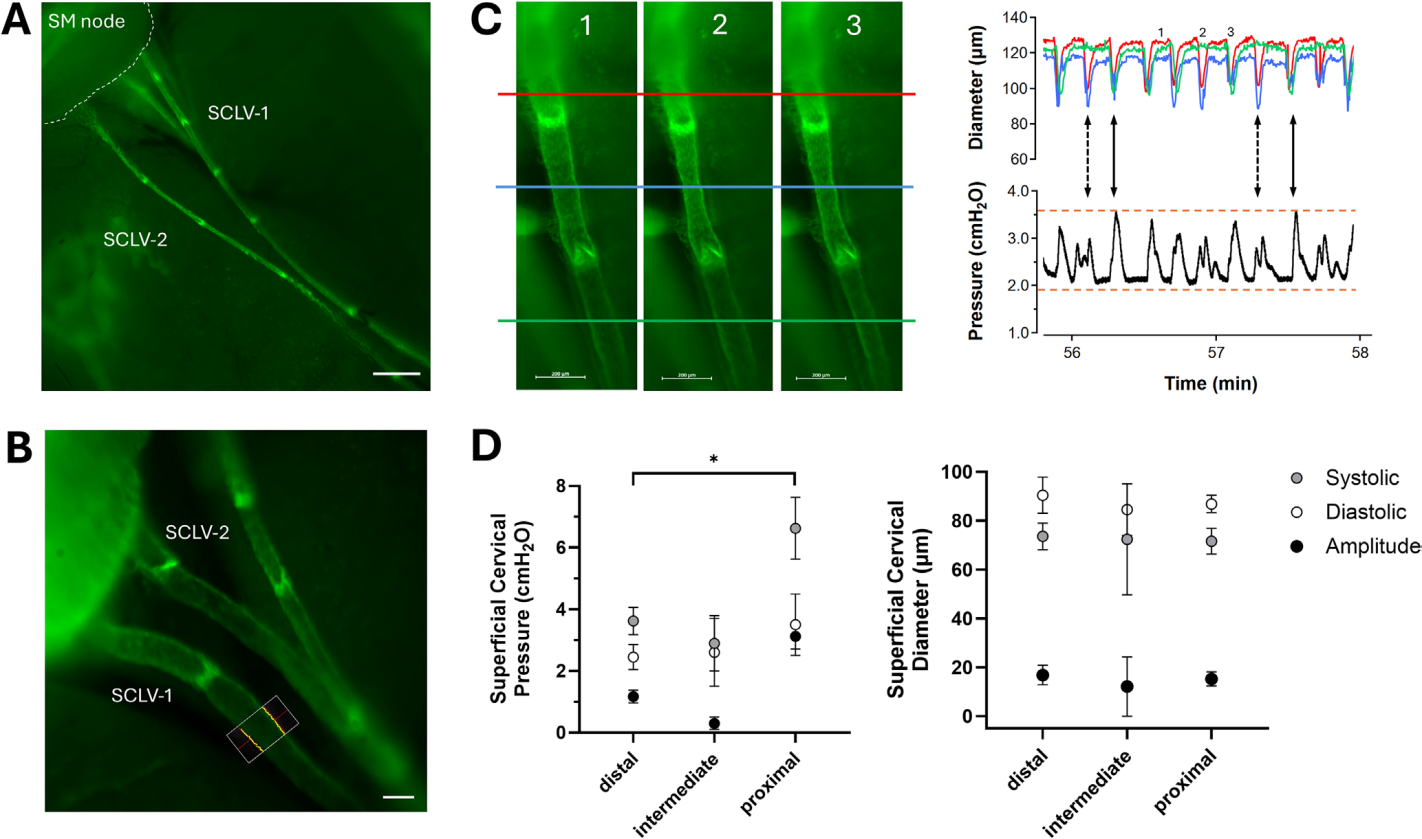
(A) Low‐magnification, composite image of the superficial cervical lymphatic collectors draining from the head to the submandibular (SM) lymph node in the neck of a *Prox1‐GFP* mouse. The image is a montage of 3 photos, each taken at a slightly different focal plane. SCLV‐1 and SCLV‐2, which drain the cranium, correspond to the numbering system used for these vessels by Koh and colleagues [32]. Scale bar = 500 μm. (B) Higher magnification view of SCLV‐1 and SCLV‐2 in another *Prox1‐GFP* mouse. This was the magnification typically used for micropuncture and diameter recordings. A diameter tracking window is superimposed on the lower portion of SCLV‐1; the yellow dots indicate the outside edge of the fluorescence signal and parallel red lines indicate the average of the detected edges for each side of the vessel wall. Scale bar = 100 μm. (C) Simultaneous servo‐null pressure and diameter recordings in a superficial cervical lymphatic vessel. Diameters were measured at the 3 sites indicated by the red, blue and green lines in the images and the measurements at those sites correspond to the red, blue and green traces on the graph. Image 1 captures the three sites during the diastolic phase of the contraction cycle. In image 2 only the middle segment exhibits a strong contraction. In image 3, the entire segment has an entrained contraction. Scale bars = 200 μm. The vertical dotted lines on the graph show blunted pressure traces corresponding to times when only the 2 proximal segments contracted, whereas the solid vertical lines indicate higher systolic pressure peaks corresponding to entrained contractions of the three segments. The upper and lower orange horizontal lines denote the maximum systolic pressure and minimum diastolic pressure, respectively, reached during the longer 6.8 min recording period from which this graph was excerpted. (D) Summary of recorded pressures and diameters in superficial cervical lymphatic vessels, showing a significant uphill pressure profile from the distal to proximal sites. *N* = 3; *n* = 10 at distal sites; *N* = 1, *n* = 2 at intermediate sites; *N* = 2, *n* = 8 at proximal sites.

Measurements were made in 16 locations within the superficial cervical lymphatic networks of five *Prox1‐GFP* mice. All vessels in all animals exhibited spontaneous contractions, although the amplitudes of contractions varied substantially from vessel to vessel and along the length of a given vessel (proximal vs. distal). In most preparations, 5–6 valves were visible over the length of each SCLV‐1 and SCLV‐2 (Figure 2A). Micropuncture was typically performed at the maximum available magnification (112×), as shown in Figure 2B, in which a diameter tracking window is superimposed near the pressure recording site. Alignment of the respective pressure and diameter traces revealed that systolic pressure peaks generally corresponded to the constriction phases of contractions, with the highest systolic pressures occurring when the contractions of multiple consecutive lymphangions were entrained (Figure 2C). Summary data for pressures recorded in SCLV‐1 and SCLV‐2 (combined) are shown in Figure 2D and grouped as follows: proximal = recorded within 1–2 valves of the submandibular node, distal = at least 5 valves from the submandibular node (generally as close to the mandible as possible), and at an intermediate site. There were no significant differences between diastolic pressures in the three regions, but systolic pressure was significantly higher in proximal vessels compared to distal vessels. Diameters and contraction amplitudes were comparable in all three vessel segments.

### 3.2 Popliteal Afferent Lymphatic Pressures

**FIGURE 3:**
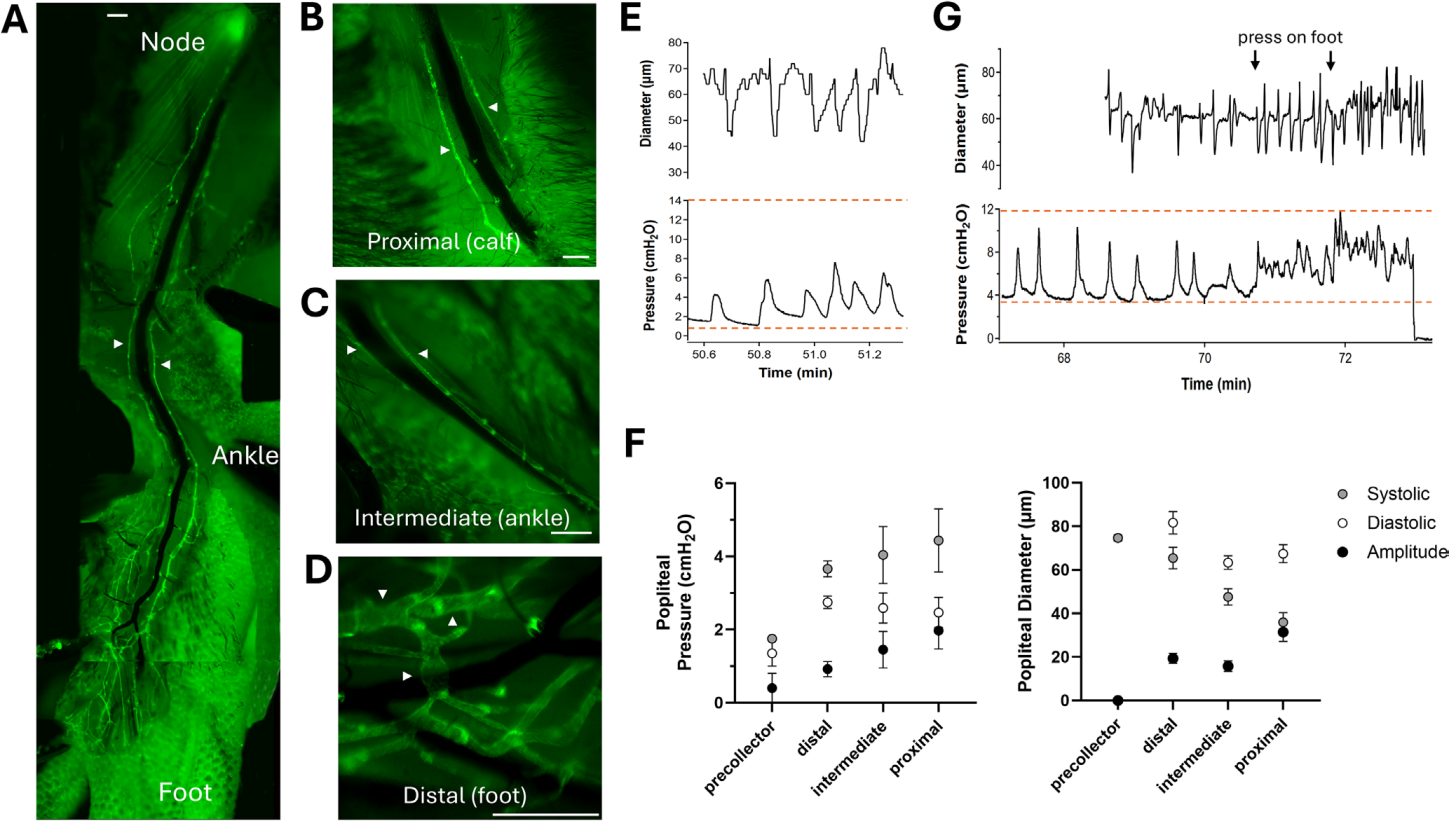
(A) Low‐magnification images (reconstructed into a montage) showing the entire length of the popliteal lymphatic network in the hindlimb of a *Prox1‐GFP* mouse. An incision was made in the skin, which was subsequently retracted and held with small clamps. Some images are not perfectly aligned because slight repositioning of the clamps and skin edges was required during imaging of the various regions. The lower part of the central (dark) saphenous vein has been enhanced for better visibility because the vein constricted during dissection and image acquisition. Arrowheads indicate lateral (left) and medial (right) popliteal collecting lymphatics. Scale bar at top = 500 μm. (B–D) Higher magnification views of the saphenous vein with the two surrounding collecting lymphatic vessels in the calf region (B), ankle region (C) and top of the foot (D). In B the lateral and medial collectors are prominent, while in C most of the lateral vessel is obscured by the saphenous vein. Arrowheads mark the lateral (left) and medial (right) popliteal collecting lymphatics. In D the vessels are a mixture of collecting vessels with localized spontaneous contractions (at arrowheads) and precollectors without spontaneous contractions. (E) Representative pressure and simultaneous diameter recording in a popliteal lymphatic collector; systolic pressure peaks correspond with constrictions, except for the last contraction. (F) Summary data for pressures and diameters in the various orders of popliteal lymphatic vessels, showing a trend for an uphill pressure profile from precollectors to the proximal collectors. (G) Pressure and diameter recordings in a popliteal collector near the ankle; at arrows, pressure is applied to the top of the foot with a cotton swab, inducing increases in diastolic pressure and frequency. Horizontal dotted lines in E and G indicate the maximum and minimum pressure fluctuations over the 11‐ and 13‐min recording periods, respectively, in these two vessels. *N* = 1, *n* = 2 for precollectors; *N* = 6, *n* = 23 at distal sites; *N* = 6, *n* = 12 at intermediate sites; *N* = 5, *n* = 12 at proximal sites.

The length of the popliteal afferent lymphatic collector from the top of the foot to the popliteal node spanned a distance of several centimeters and encompassed a total of 20–25 intraluminal valves (Figure 3A). Pressure recordings were made in 12 proximal segments of two C57Bl/6 and three *Prox1‐GFP* mice, 13 intermediate segments of six *Prox1‐GFP* mice, and 20 distal segments of six *Prox1‐GFP* mice. In proximal segments (Figure 3B) and intermediate segments (Figure 3C), contractions occurred in most collecting vessels, and pressure measurements were made only in the two main collectors. The networks in the foot (Figure 3D) contained a mixture of precollectors (without spontaneous contractions) and collectors (with contractions typically localized to only a few sites along a given vessel), suggesting an incomplete coverage of lymphatic muscle cells. Collectors in this region tended to be larger than in more proximal regions. Precollectors displayed a wide range of diameters (some < 20 μm, as in a vessel near the top of Figure 1A), and pressures were only successfully measured in a few of the larger ones. The typical popliteal collector constricted strongly during micropuncture, after which diastolic pressure was elevated for several minutes before declining over the subsequent 1–5 min as diameter returned to its original value. The stable values were then averaged over several contraction cycles for measurements of systolic/diastolic pressures and diameters. A representative recording from an intermediate popliteal segment is shown in Figure 3E, in which constrictions again strongly corresponded to the systolic pressure peaks. Both systolic and mean pressures tended to increase moving from precollectors (mean P_sn_ = 1.5 cmH_2_O) to the most proximal collecting lymphatic vessels (mean P_sn_ = 3 cmH_2_O), as shown in Figure 3F, but the differences in pressures along the lengths of the popliteal collectors did not reach statistical significance. Contraction amplitude was zero in the precollectors and highest in the most proximal segments. In two experiments, gentle pressure was applied to the top surface of the foot while recording pressure in a popliteal collector near the ankle (Figure 3G). This rapid rise in recorded pressure served to confirm luminal placement of the servo‐null pipette tip but also resulted in a rapid rise in diastolic pressure and contraction frequency, consistent with what has been observed in popliteal lymphatics ex vivo [22, 38, 39].

### 3.3 Mesenteric Network Pressures

**FIGURE 4:**
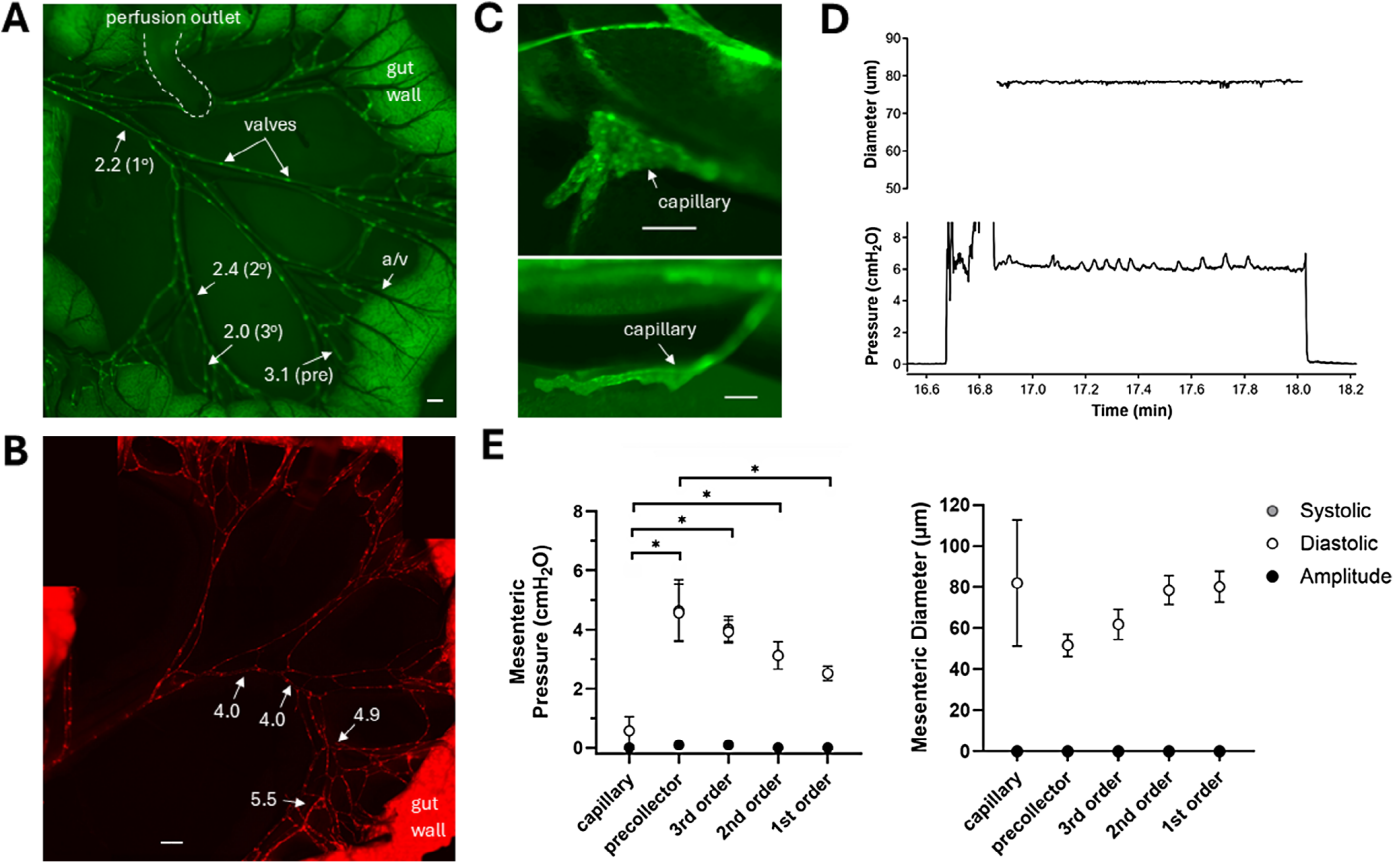
(A) Low magnification view of the mesentery of the terminal ileum in a *Prox1‐GFP* mouse, showing the extensive networks of collectors and precollectors draining the gut wall (right side) toward the mesenteric lymph node (out of field of view on left side). Numbers correspond to pressures measured in specific vessels marked by arrows. Scale bar = 500 μm. (B) Low magnification view of a similar region in a *Prox1‐tom* mouse, with numbers corresponding to measured pressures. Some edges of the gut wall are blacked out. Scale bar = 1 mm. (C) Images of initial lymphatic capillaries near the edge of the gut wall (top) and a vein (bottom). Scale bars = 200 μm. (D) Recording of pressure in one of two mesenteric collectors in which pressure pulsations are evident, but the pulsations do not correspond to changes in diameter. (E) Summary data for pressures and diameters in the various orders of lymphatic vessels in the mouse mesentery, showing a significant downhill pressure profile from precollectors to first order collectors. *N* = 3, *n* = 4 for capillaries; *N* = 4, *n* = 6 for precollectors; *N* = 8, *n* = 15 for third order collectors; *N* = 9, *n* = 10 for second order collectors; *N* = 8, *n* = 12 for first order collectors.

Pressure measurements were made in mesenteric lymphatic networks of 10 *Prox1‐GFP* mice, two *Prox1‐tom* mice, and two WT mice. A low‐magnification image of the mesenteric network in the terminal ileum of a *Prox1‐GFP* mouse is shown in Figure 4A, with micropuncture location sites and corresponding hydrostatic pressure measurements stated. Pressure was higher in the third order collector than in the second order collector, and lowest in the first order collector. A slightly lower pressure was recorded in another third order collector near the gut wall. This was a pattern observed in several mesenteric preparations, where vessels in some regions had relatively low pressures compared to vessels in the surrounding network. A slightly different pattern is illustrated in Figure 4B in the mesenteric network in the terminal ileum of a *Prox1‐tom* mouse. In this example, pressures were consistently higher near the gut wall and declined sequentially as the collectors coalesced and exited the mesentery. Higher magnification views of two mesenteric capillaries (with widely varying diameters) are shown in Figure 4C.

There were no obvious spontaneous contractions of mesenteric collecting vessels in any mouse, and P_sn_ recordings in those vessels were almost always non‐pulsatile; in the only two exceptions (one shown in Figure 4D), small irregular changes in pressure occurred in synchrony with gut wall movement but without any corresponding vessel diameter changes, suggesting that the pulsations were transmitted from the gut wall. Summary plots showing average pressures and diameters for the various orders of collecting vessels, precollectors, and a few (*n* = 4) capillaries are shown in Figure 4E. Unlike the superficial cervical and popliteal networks, pressures in the mesentery *declined* moving away from the gut wall toward the more proximal vessels. An exception was that P_sn_ measurements in mesenteric lymphatic capillaries were significantly lower than in the precollectors draining the gut wall. Those measurements were difficult to make as the initial lymphatics were almost always embedded under fat, which interfered with positioning of the pipette tip and transiently interrupted electrical continuity of the servo‐null circuit. Consequently, only a few successful measurements could be made in initial lymphatics that were found adjacent to mesenteric veins or near the gut wall (Figure 4C). No successful measurements were made in gut wall capillaries due to movement, even after attempts to suppress gut wall motility.

## 4 Discussion

### 4.1 Summary

Hydrostatic pressures in collecting lymphatic vessels of the mouse were comparable to those in the rat [6, 12, 40, 41] but lower than those recorded in some larger species [15, 17]. Pulsatile pressures were recorded only in lymphatic networks in which collecting vessels exhibited spontaneous contractions. In such networks, both systolic and diastolic pressures increased moving from more peripheral vessels toward the first lymph node, in agreement with previous observations [3, 4]. This “uphill” pressure profile was more consistent (but not statistically significant) in the popliteal network than in the superficial cervical network, presumably because the pressure profile in the former could be measured over much longer distances (25 vs. 5 lymphangions). Indeed, numeric modeling studies predict uphill pressure profiles in series‐coupled networks of actively pumping lymphangions [42, 43, 44]. In contrast, the measured pressure profile in the mesenteric lymphatic network was “downhill,” unlike that previously recorded in rat mesentery (in which spontaneous contractions in collectors are robust [4]). This is the first such finding and it suggests that, in the absence of active lymphatic contractions, net lymph transport in the mesentery and other such networks occurs only during a favorable luminal pressure difference. Low pressures in the initial lymphatic capillaries of the mesentery suggest, under the conditions of our experiments (in which interstitial pressures are near zero in an open preparation [5]), that the vessels draining the mesentery per se are not transporting lymph. Presumably, these mesenteric capillaries become absorptive and their intraluminal pressures increase in states such as ascites when interstitial pressures are elevated.

### 4.2 Technical Improvements

To our knowledge, previous servo‐null pressure studies have been made only under brightfield illumination, conditions under which lymphatic vessels in many or most regions are hard to visualize. Lymphatic vessels are visible in the bat wing, but only after that tissue has been artificially hydrated to expand the vessels and stimulate their contractile activity [9]. Lymphatic vessels can be visible under brightfield illumination in the mesentery of young animals, or when the network is filled post‐prandially with lipid [45], but many vessels become obscured by fat as the animals age. Other lymphatic networks can be visualized after tracer injection into distal portions of the network, but doing so can alter the baseline pressures/flows [28]. For these reasons, fluorescent reporter mice are advantageous, allowing for visualization of entire lymphatic networks in their “normal” state, as illustrated by the images in Figures 3A and 4A,B and the elegant mapping studies of diaphragmatic lymphatic networks in *Lyve1‐GFP* mice by Negrini [11]. However, visualizing and guiding a servo‐null pipette tip for successful micropuncture was found to be much more difficult under fluorescence than brightfield illumination. New methods were therefore devised to record servo‐null pressures in lymphatic vessels of fluorescence reporter mice, to formulate a set of criteria for valid pressure recordings made under those conditions (see Methods), and to simultaneously measure lymphatic pressures and diameters in vivo, which to our knowledge has only been performed in a single, previous study [6]. These techniques should be useful to others who wish to pursue similar studies in mouse models of lymphatic disease, including primary and secondary lymphedema, lymphatic network dysplasia, and lymphatic valve defects.

### 4.3 Superficial Cervical Lymphatic Network

Superficial cervical lymphatic collectors are now being actively studied after renewed appreciation of the lymphatic networks in and around the brain [46, 47, 48] and their possible roles in the clearance of brain metabolic products [49, 50]. Superficial cervical collectors actively contract [32, 51] whereas deep cervical collectors have infrequent or no contractions [52]. The high visibility of the superficial cervical vessels (and the in‐focus views of multiple series‐coupled segments in some animals) allowed us to measure diameters of consecutive collecting vessel segments simultaneously with pressure. Diastolic pressures were ~3 cmH_2_O, with contraction amplitudes ~15 μm (equivalent to a normalized amplitude of ~18%, but with that estimate limited by lack of measurement of the true maximum passive diameter in vivo). Pressures in these vessels presumably vary in proportion to the volume of the cerebral ventricles. The amplitudes of ~30% recorded in recent ex vivo studies of superficial cervical collectors [32, 51] can be achieved only at pressures lower (1–2 cmH_2_O) than we observed in vivo.

### 4.4 Popliteal Lymphatic Network

Systolic pressure peaks corresponded most often, but not always, to active contractions (Figure 3E), and the highest peaks in systolic pressures occurred when several consecutive lymphangions contracted in phase. In other cases, diastolic pressures were ~2 cmH_2_O during active contractions but declined to 0.5 cmH_2_O if long (> 3 min) pauses occurred (not shown), suggesting that pressures in the upstream capillary network are ≤ 0.5 cmH_2_O. Intraluminal pressures and the associated frequencies of pulsatile pressures were sensitive to gentle pressure applied to the top of the foot with a cotton swab. This crude intervention no doubt compressed both distal capillaries and collectors, and the rapid rise in P_sn_ measured in Figure 3G is consistent with the idea that pressures are transmitted rapidly through the collecting vessel network. The highly variable contraction amplitudes (and pressure peaks) recorded herein contrast with the nearly uniform amplitude contractions routinely observed in ex vivo studies; however, in such studies, only small pressure spikes occur in systole when inflow and outflow pressure levels are set to equal values [10, 34, 53, 54].

### 4.5 Mesenteric Lymphatic Network

Pressure profiles in mouse mesenteric lymphatic networks were opposite to those previously published for the rat mesentery [4]. However, mesenteric collectors in the mouse are unique in *not* exhibiting spontaneous, propulsive contractions [22] and therefore are likely not representative of the mesenteric networks in other species, except in the post‐prandial state when active contractions in other species are inhibited [45]. Flow is apparently driven down a passive luminal pressure gradient in the mouse mesentery, implying that gut wall capillary pressure (which we could not measure) is > 4.5 cmH_2_O. Although we made no attempt to control the timing of our experiments relative to feeding of the mice, this estimated value is comparable to villus capillary pressures measured by Lee in a simulated post‐prandial reabsorption state [7].

### 4.6 Perspectives

These measurements provide information on hydrostatic pressures in three well‐studied lymphatic networks of normal, healthy mice. They establish that a “downhill” pressure profile occurs in the absence of active lymphatic contractions, while “uphill” pressure profiles develop in lymphatic networks exhibiting active contractions of collecting vessels. These findings also suggest that baseline pressures of 0.5–3 cmH_2_O are appropriate for simulating the physiological diastolic pressure range in ex vivo studies of cannulated lymphatic collectors from mice. In the future, similar measurements will be required in models of lymphatic disease to ascertain if, and to what extent, lymphatic pressures may be altered in ways that detrimentally impact net lymph transport.

## Funding

This work was supported by National Institutes of Health grant R01 HL‐122578 to Michael J. Davis.

## Conflicts of Interest

The author declares no conflicts of interest.

## Data Availability

All data needed to evaluate the conclusions in the paper are present in the paper. Data will be made available from the author upon reasonable request.
